# Impact of routine assessment of health-related quality of life coupled with therapeutic information on compliance with endocrine therapy in patients with non-metastatic breast cancer: protocol for a randomized controlled trial

**DOI:** 10.1186/s13063-020-04397-w

**Published:** 2020-06-16

**Authors:** Ariane Mamguem Kamga, Cyril Di Martino, Amelie Anota, Sophie Paget-Bailly, Charles Coutant, Patrick Arveux, Isabelle Desmoulins, Tienhan Sandrine Dabakuyo-Yonli

**Affiliations:** 1 Epidemiology and Quality of Life Research Unit, INSERM U1231, Georges Francois Leclerc Centre – UNICANCER, 1 rue du Professeur Marion, Dijon Cedex, 21000 France; 2 Georges François Leclerc Centre – UNICANCER, 1 rue du Professeur Marion, Dijon, 21000 France; 3 Methodological and Quality of Life Unit in Oncology, University Hospital Of Besançon, 3 Boulevard Alexandre Fleming, Besançon, 25000 France; 4 INSERM, EFS BFC, UMR1098, Interaction Hôte-Greffon-Tumeur/Ingénierie Cellulaire ET Génique, University Bourgogne Franche-Comté, 32 Avenue de l’Observatoire, Besançon, 25000 France; 5 National Quality of Life and Cancer Platform, 1 rue du Professeur Marion, Dijon, 21000 France; 6 Surgery Department, Georges François Leclerc Centre – UNICANCER, 1 rue du Professeur Marion, Dijon, 21000 France; 7 Burgundy Franche-Comté University, 7 Boulevard Jeanne d’Arc, Dijon, 21000 France; 8 Centre for Research in Epidemiology and Population Health (CESP), INSERM U1018, University Paris-Sud, UVSQ Gustave Roussy, 12 Avenue Paul Vaillant Couturier, Villejuif, 94800 France; 9 Medical Oncology Unit, Centre Georges-François Leclerc Centre – UNICANCER, 1 rue du Professeur Marion, Dijon, 21000 France

**Keywords:** Breast cancer, Health-related quality of life, Compliance, Therapeutic information, Endocrine therapy

## Abstract

**Background:**

Despite its proven efficacy in reducing recurrence and improving survival, adherence to endocrine therapy (ET) is suboptimal in women with breast cancer (BC). Health-related quality of life (HRQoL) in BC has been widely studied and many positive effects have been highlighted. Recently, a link between HRQoL and compliance with ET has been suggested, which would suggest a potential role for HRQoL assessment in improving compliance with ET. With the advent of digital technologies, electronic collection of HRQoL on a tablet is now possible. Thus, we hypothesize that systematic HRQoL assessment (using a tablet, prior to each consultation, with presentation of scores to clinicians) coupled with therapeutic information could have an impact on 12-month compliance with ET in patients with non-metastatic BC.

**Methods:**

In this study, we will include 342 women with non-metastatic hormone receptor–positive BC with an indication for treatment with ET. Patients will be randomly assigned 1:1 by minimization and stratified by age, stage, type of ET prescribed, and presence of comorbidities (or not) in two arms. The intervention will consist of numerical HRQoL assessment using the CHES (Computer-based Health Evaluation System) software before each consultation (with delivery of scores to clinicians) coupled with therapeutic information. Therapeutic information will consist of three workshops related to understanding the prescription, nutrition, and fatigue. A reminder letter will be sent to patients every month. Patients in the control group will follow standard care. HRQoL will be assessed using a classic “paper-pencil” collection at baseline in both arms to ensure comparability between arms and at 12 months. The primary endpoint is 12-month compliance with ET. Patient satisfaction with care and the clinicians’ perception of the usefulness of routine HRQoL assessment will also be assessed.

**Discussion:**

This study will allow clinicians to identify and better understand the areas in which patients who receive ET have difficulties and thus it will assist clinicians with patient management. Systematic evaluation of HRQoL could provide an additional endpoint for measuring patients’ health status and treatment-related symptoms, including ET. If the results of this study are positive, this intervention could be proposed as an integral part of daily clinical practice in patients who receive ET.

**Trial registration:**

ClinicalTrials.govNCT04176809. Registered Nov. 25, 2019.

## 1. Background

Currently, a large number of hormonal and cytotoxic oral therapies are available for breast cancer (BC) management. The major advantage of these therapies is their convenience due to self-administration [1, 2]. Currently, in cancer treatment, more than 40 oral drug specialties are reimbursed by the French health insurance system [1]. These oral forms present a new challenge in oncology, namely concerning treatment compliance. Compliance is defined as the extent to which the patient follows the prescriber’s recommendations [3]. Oral anticancer therapies raise concerns about poor compliance, particularly the impact of adverse effects due to non-compliance on clinical outcomes. Non-compliance compromises overall efficacy of oral therapies and potentially leads to treatment failure [4].

In BC, the oral form of endocrine therapy (ET) is by far the most widely investigated in terms of compliance [1]. Compliance with oral ET is not optimal in BC. Indeed, only 59% of women who receive ET for BC remain compliant one year after prescription [5] and this percentage ranges between 41% and 72% at the end of 5 years of treatment [6]. Poor compliance with ET is associated with decreased survival [7, 8], increased risk of recurrence [7], and poor prognosis [9]. To improve compliance, several studies [10–12] have implemented interventions via educational materials (letters, information booklets, and telephone interviews) but none has shown a significant effect. Hadji et al., in their study testing the addition of educational materials to standard therapy versus the standard therapy only in women taking anastrozole, have shown no significant effects on compliance and persistence with adjuvant anastrozole [10]. In a study by Ziller et al., patients receiving additional/supplemental information appeared to have an improved adherence rate, even though differences between groups with regard to the primary endpoint were not statistically significant [11]. In another study, postmenopausal women with hormone receptor–positive (HR^+^) early-stage BC treated with aromatase inhibitors (AIs) were randomly assigned to one of two groups: one receiving only AIs and the other one receiving educational materials plus AIs. This study has shown that education materials do not improve compliance in this patient population and has highlighted the complex nature of compliance and persistence [12].

It is interesting to note that compliance is a multidimensional phenomenon that can result from various factors related to patients (social support, patient beliefs, and psychosocial factors), treatments (side effects), or health-care systems (poor patient–health-care provider relationship) [13].

**Fig. 1:**
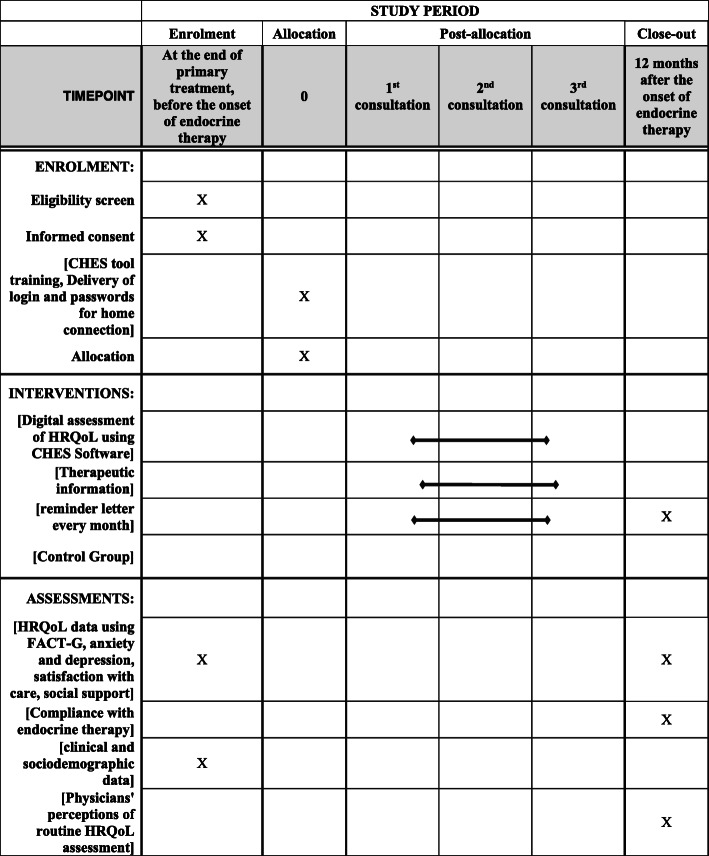
Schedule of enrolment, interventions, and assessments

Evaluation of the quality of care is of major importance, but to date, most quality initiatives have focused on assessing adverse events, clinical processes, and cost variables. Less attention has been given to indicators of clinical improvement measured from the point of view of the patient [14]. To give patients the opportunity to express their perception concerning their own health in clinical trials and routine practice, it is necessary to plan and assess the use of health-related quality of life (HRQoL) in clinical research and practice (Fig. 1).

HRQoL is gathering increasing interest in oncology, as reflected by the number of scales being developed and the increased use of HRQoL measurement as an outcome in randomized controlled trials. Reasons for this growing interest may be related to significant treatment toxicities but also to the fact that HRQoL has been shown to be a prognostic factor for survival [15, 16]. In many cases, symptom palliation of and improvement of HRQoL, rather than increased survival, are the main goals of treatment. Thus, the American Society of Clinical Oncology and the US Food and Drug Administration recommend HRQoL as the second endpoint after overall survival and the primary endpoint of treatment efficacy if treatment has no effect on overall survival. Over the past decade, HRQoL in BC has been widely studied and many determinants have been identified [17, 18].

Routine evaluation of HRQoL was shown to have a positive impact on communication between patients and medical staff [19] and on clinical parameters such as the duration of treatment without relapse and survival [20, 21]. Recently, it has been suggested that there may be a link between HRQoL and compliance with oral ET. Indeed, Pinheiro et al. reported that women with poorer HRQoL were more likely at risk of non-adherence [22] in a study addressing the association between HRQoL and under-use of ET in women with hormone-sensitive BC. This result suggests that focusing on a modifiable factor such as HRQoL could be a way to improve compliance with oral ET. In addition, to date, the modulatory effect of HRQoL on compliance with oral treatments in BC has not been studied.

With the advent of digital technologies, it is now possible to perform HRQoL measurements using a tablet with instantaneous score generation [23]. This mode of measurement provides real-time results to clinicians (through graphics) and generates alerts (color codes) if HRQoL scores are deteriorated, allowing adjustment of patient support in a personalized way. From the patient’s point of view, the inclusion of numerical assessment in the process of data collection has shown benefits in disease management and symptom control [24], improving survival [20] and patient-clinician communication [19]. Moreover, it potentially facilitates clinical decision making through the systematic and continuous collection of symptom data [25].

Since compliance is a multidimensional phenomenon, any attempt to improve it should encompass several potential contributors. Thus, we hypothesize that electronic measurement of HRQoL (before each consultation with scores communicated immediately to clinicians who can discuss it with patients), coupled with therapeutic information, will improve 12-month compliance rate with ET in patients with non-metastatic BC. At a later step, we will assess the role of social support and psychological distress as potential modulators of compliance with ET, the capacity of HRQoL to predict compliance with ET, patient satisfaction with care, and physician perception regarding the clinical utility of routine HRQoL evaluation.

## 2. Methods

To achieve study aims, we will carry out a randomized, interventional, prospective study. Patients will be recruited at the Dijon cancer center (Georges François Leclerc-Unicancer Centre) in France. This center is specialized in cancer management; therefore, clinicians are experienced in patient HRQoL evaluation and are accustomed to take this criterion into consideration in routine patient management.

Women who are at least 18 years old and have non-metastatic HR^+^ BC will be included in this study. These women will have to be at the end of primary treatment, have an indication for ET treatment during 5 to 10 years, be affiliated with a French social security scheme or the beneficiary of such a scheme, and have agreed to participate by providing written consent. Patients who participate in another clinical trial where HRQoL is assessed will not be included, nor will women for whom HRQoL evaluation is not possible (cognitive disorders, psychiatric disorders, and people who do not speak French).

### 2.1. Inclusion procedure

The study will be proposed to eligible patients by their doctors (oncologists, surgeons, and radiation oncologists). Patients will be included at the time of the first prescription of ET (at the end of the treatments by surgery with or without chemotherapy with or without radiotherapy). Once included, patients will be randomly assigned to one of the two study arms (interventional arm or control arm). Patients then will be referred to the clinical research associates, whose role will be to collect patient data (clinical and sociodemographic) in both study arms and to instruct patients in the interventional arm on how to use the CHES (Computer-based Health Evaluation System) software [26]. For patients who wish to access the platform from home, a username and password will be provided. If needed, clinical research associates will assist patients to complete HRQoL questionnaires. This information will be collected in the case report form to take into account social desirability bias.

### 2.2. Randomization

Eligible patients who agree to participate will be randomly assigned to one of two parallel arms (ratio 1:1) by the minimization technique with stratification by age, stage, presence or absence of comorbidities, and type of ET prescribed.

### 2.3. Intervention arm

#### 2.3.1. Assessment of HRQoL

The intervention will consist of an electronic measurement of patient HRQoL before each consultation with delivery scores to clinicians, who can discuss it with patients and couple it with therapeutic information. Before their consultation, patients will complete the EORTC-QLQ-C30 and the EORTC-QLQ-BR23 questionnaires via a touch pad or from their home via a secure web portal. Patients will complete the questionnaires via the CHES software [26]. The CHES software was developed by ESD (Innsbruck, Austria) in collaboration with the EORTC Quality of Life Group to facilitate the inclusion of HRQoL measurement instruments in research projects and daily clinical practice. This mode of collection provides clinicians with real-time results and generates alerts in the event of clinically significant deterioration of patients’ HRQoL scores, allowing them to tailor treatment. The scores then will be generated and provided to patients and clinicians in a graphic form, describing scores course. Access to the CHES web portal will be open to patients outside consultation time points to enable them to monitor HRQoL if necessary. In case of HRQoL minimal impairment (a decrease lower than 20 points) [27], self help tools can be generated, providing patients with indications on how to deal with certain adverse effetcs. In the other hand, in case of significant impairment of HRQoL (a decrease higher than 20 points) [27], the clinical research assistant will receive an alert and if, necessary, she will the information to the medical team for appropriate care.

#### 2.3.2. Therapeutic information

Therapeutic information will consist of workshops on various themes and will be coordinated by a pharmacist in charge of therapeutic education at the Dijon cancer center. This health-care professional will be responsible for the organization and implementation of these workshops. Workshops will be performed by pharmacists, nurses, or dieticians. Only at workshop 1 will attendance be required; other workshops will be optional.

Workshop 1 will deal with “understanding the prescription”. The aim is to inform patients of their ET and treatment benefits. It also helps patients to recognize and react to the occurrence of possible adverse effects and anticipate their occurrence through appropriate preventive means. This workshop will be held within 2 months after the first prescription of ET and will be performed by pharmacists with training in therapeutic education.

Two additional optional workshops on nutrition (workshop 2) and fatigue (workshop 3) will be offered. These workshops will be collective and performed by dieticians and nurses, respectively. Workshop 2, on nutrition, will focus on the benefits of exercise and the need to adopt an appropriate diet. Patients will have the opportunity to express their representations and experiences related to their diet and the consequences of disease and treatments on diet. Workshop 3, on fatigue, will address the recognition of fatigue and early management of this symptom. Patients can describe their experiences of fatigue and how their life is affected by it. Moreover, they can identify possible causes and finally discuss solutions to better this symptom.

Every month, a letter encouraging patients to regularly take their medication will be sent. This letter, derived from the work of Hadji et al. [10], will include some tips on how to deal with some particular side effects of ET.

#### 2.3.3. Control arm

Patients in the control arm will receive standard care. They will not undergo digital HRQoL collection, and therapeutic information workshops will not be proposed. This information will be taken into account when performing statistical analyses.

For both arms, HRQoL will be evaluated at baseline using the FACT-G (Functional Assessment of Cancer Therapy - General) questionnaire to ensure the comparability of groups concerning HRQoL at inclusion and again at 12 months. This HRQoL assessment will be performed using a traditional paper questionnaire, and scores will not be provided to clinicians. Anxiety and depression, social support, and patient satisfaction with care will also be assessed in both arms at baseline and 12 months after.

### 2.4. Endpoints

#### 2.4.1. Primary endpoint

The primary endpoint of this study is 12-month compliance with ET as evaluated using the Morisky Green Levine (MGL) scale. Patients will be considered to be compliant if they have a high adherence in the MGL scale.

#### 2.4.2. Secondary endpoints

The secondary endpoints will be anxiety and depression assessed by the Hospital Anxiety and Depression Scale (HADS) questionnaire, social support assessed by Sarason’s Social Support Questionnaire (SSQ6) questionnaire, and HRQoL assessed by the FACT-G questionnaire. HRQoL data using the FACT-G questionnaire will be assessed at inclusion in both arms to ensure comparability between groups and at 12 months to assess the predictive value of HRQoL on compliance with ET. Patient satisfaction with care will be assessed by the EORTC-SAT-C33 and EORTC-OUT-PATSAT-7 questionnaires. Physician perception regarding the utility of systematic HRQoL evaluation will be assessed using an ad-hoc questionnaire derived from the work of Velikova [19, 28], including the perceived utility and satisfaction of routine assessment HRQoL, reasons for use/non-use, and the intention to adopt this assessment in routine care.

#### 2.4.3. Sample size

A total of 342 patients with non-metastatic BC are required, based on the following assumptions: an estimated compliance rate of 59% [5] in patients with non-metastatic BC one year after the first prescription of ET, a bilateral alpha of 5%, and statistical power of 80%; to show a 15% difference in compliance between patients in the intervention arm and those in the control arm 1 year after the first prescription, it is necessary to include 155 patients per arm (bilateral chi-squared test). Taking into account a loss to follow-up rate of 10%, a total of 171 patients will be included in each arm (nQuery Advisor V7).

#### 2.4.4. Data collected

Sociodemographic characteristics (age, sex, level of education, family situation, socio-professional category, and work time), medical and surgical history, date of tumor diagnosis, tumor characteristics, previous treatments, patient clinical characteristics at inclusion and at each follow-up visit (weight, height, and overall patient condition), concomitant treatment, type of ET received, HRQoL data (FACT-G), anxiety and depression (HADS), social support (SSQ6), treatment modification (change in the type of ET), treatment-related toxicities, and their grade will be collected. Patient satisfaction with provided care will be measured using the EORTC-SAT-C33 and the EORTC-OUT-PATSAT-7. Clinician perceptions of systematic HRQoL assessment (utility and perceived satisfaction, reasons for use or non-use, and the intention to adopt it in practice) will be collected via an ad-hoc questionnaire. Sociodemographic data and reasons for refusing to participate will be documented for patients who refuse to participate. Data on patient withdrawal or death will be documented in the case report form. Reasons for study withdrawal should also be documented. All data from this study will be transcribed in an electronic case report form (Clinsight).

### 2.5. Questionnaires and tools

#### 2.5.1. EORTC-QLQ-C30 and EORTC-QLQ-BR23 questionnaires

The cancer-specific EORTC-QLQ-C30 questionnaire and its BC-specific QLQ-BR 23 module are available in French and are validated tools to assess HRQoL in cancer and more specifically in BC [29]. The EORTC-QLQ-C30 questionnaire makes it possible to assess one dimension of HRQoL/overall health, five functional dimensions (physical, current activities, cognitive, emotional, and social), eight dimensions of symptoms (fatigue, nausea and vomiting, pain, dyspnea, insomnia, loss of appetite, constipation, and diarrhea), and a scale of financial difficulties. The EORTC-QLQ-BR23 consists of 23 questions which assess four functional dimensions (body image, sexual activity, sexual pleasure, and future perspectives) and four dimensions of symptoms (side effects of systemic therapy, breast symptoms, symptoms at the arm, and upset by hair loss). Scores are generated per dimension in accordance with the EORTC [30] scoring rules. These scores vary from 0 (worst) to 100 (best) for the functional and global health parameters and from 0 (best) to 100 (worst) for symptom parameters.

#### 2.5.2. MGL questionnaire

The four-item MGL Medication Adherence Scale was developed by Morisky et al. [31] to measure adherence to treatment. It has a range of 0 to 4, where 0 is very low and 4 is highest. Patients are categorized according to three levels of adherence: high (score equal to 4), moderate (score equal to 2 or 3), and low (score equal to 0 or 1).

#### 2.5.3. HADS questionnaire

The HADS is an instrument for detecting anxiety and depressive disorders. It was validated and adapted in French by Lepine et al. [32] in 1989. This scale has 14 items rated from 0 to 3 and covers two dimensions. Seven questions are related to the anxiety dimension and seven are related to the depressive dimension, yielding two scores: A (anxiety) and D (depression). The maximum score for each dimension is 21. A score of 11 or higher indicates the probable presence of the disorder.

#### 2.5.4. FACT-G questionnaire

The FACT-G is a 27-question tool validated in patients with cancer and has four subscales to assess well-being. The FACT-G instrument assesses four HRQoL domains: physical well-being (seven items), social or family well-being or both (seven items), emotional well-being (six items), and functional well-being (seven items). Respondents use a 5-point Likert-type scale which rates the relevant domain from 0 (not at all) to 4 (very much). From these subscales, a global score is obtained [33, 34]. The FACT-G total score varies from 0 to 108. The higher the score, the better the HRQoL.

#### 2.5.5. EORTC PATSAT-C33 and EORTC-OUT-PATSAT-7 questionnaires

The EORTC-PATSAT-C33 questionnaire and its EORTC-OUT-PATSAT7 ambulatory context-specific supplementary module were developed to assess perceptions of patients with cancer regarding the quality of care received [35]. They consist of 33 and 7 items, respectively. The EORTC-PATSAT-C33 questionnaire includes three sections on the perceived quality of care provided by physicians, radiotherapy nurses/technicians, and services/care organization. The clinician section includes three dimensions that address technical skills (three items), quality and quantity of information exchanged (three items), and behavior (four items). The radiotherapy nurses/technicians section has two dimensions: information provision and reactivity (three items) and affective behavior (four items). The service and organization of care section has three dimensions: coordination (four items), interaction with the health-care team (seven items), and five single items.

The complementary EORTC-OUT-PATSAT7 includes two dimensions dealing with convenience of care (three items) and transition (three items) and a single item on continuity of care. The instructions invite patients to evaluate the most recent care experience and to specify the cancer care setting. The score varies from 0 to 100. A higher score indicates higher level of satisfaction.

#### 2.5.6. SSQ6 questionnaire

Social support will be evaluated by the SSQ6 questionnaire, validated and adapted in French by Rascle et al. in 2005 [36]. This questionnaire reflects the support available in patients’ environment. Social support is measured across two dimensions: support availability, through the number of contacts that the patient can count on (0 to 9 people), and quality of support, through patient satisfaction with support received.

Each item represents a situation in which the patient may need support. The patient is asked to cite the number of people that she could count on in that particular situation. Concerning the second item, the patient is asked to assess satisfaction with the support provided. The scores are generated in accordance with Sarason’s recommendations. A score is calculated for each dimension. The support availability score is calculated as the sum of the number of people available for the six items; this score ranges from 0 to 54, with 54 representing the highest availability. The social support satisfaction score is calculated by the sum of the satisfaction of the six items; this score ranges from 6 to 36, with 36 representing the highest level of satisfaction [37].

#### 2.5.7. Ad-hoc questionnaire

This ad-hoc questionnaire, derived from the work of Velikova and colleagues [19, 28], will assess the perception of doctors concerning the interest of systematic HRQoL evaluation. It evaluates the following criteria: usefulness of the systematic evaluation of HRQoL, perceived satisfaction with systematic evaluation of HRQoL, reasons to use it (or not) in current clinical practice, and whether to adopt it routinely.

#### 2.5.8. CHES

For this study, a French version of the CHES web-based solution will be available. Each patient will be represented by an identifier to guarantee data confidentiality and anonymous exploitation of the database for statistical purposes. CHES offers a cross-sectional presentation (presentation of scores for each dimension) and a presentation of the longitudinal course of HRQoL for each dimension of the questionnaires. CHES also minimizes input errors, reduces the number of missing data related to data collection, and avoids ambiguities in the responses. This software also allows remote access via a platform that will let patients (outside the consultations) give an input on their HRQoL in the event of a deterioration or improvement perceived at the clinical level.

#### 2.5.9. Follow-up

Patients who receive adjuvant ET will attend a consultation every 6 months, and HRQoL assessment will be performed at each visit. As the aim of this project is to integrate this intervention in daily clinical practice, it will be performed in agreement with patients’ routine follow-up. Patients will complete the questionnaires prior to each consultation, either at home via access to the web portal within 24 h before consultation or at the time of consultation. During consultation, the physician will have access to the results immediately, via the secure web portal, and will be able to discuss them with the patient.

### 2.6. Statistical analyses

#### 2.6.1. Descriptive analysis

A descriptive analysis of patients’ clinical and sociodemographic characteristics at inclusion will be performed for each arm. Data will be expressed as number and percentage for categorical variables and as mean ± standard deviation or as median and interquartile range for continuous variables, as appropriate. The number of missing data will be specified. The number of patients with available data will also be specified for quantitative variables. Normality will be tested by using the Shapiro–Wilk test. According to data distribution, independent Student *t* tests or non-parametric Mann–Whitney tests will be used to compare results between groups. The basic categorical characteristics will be compared using tests of chi-squared or Freeman Halton according to the number of the variable categories. Continuous variables can be transformed into categorical variables according to thresholds defined by the literature.

#### 2.6.2. HRQoL analysis

All HRQoL scores will be calculated according to FACT-G guidelines and described according to the arm (interventional arm or control arm). A logistic regression model will be used to assess the capacity of HRQoL to predict 12-month compliance with ET. The modulatory potential of social support on compliance will be assessed using an interaction term between the availability/satisfaction of social support that patients receive and HRQoL in a logistic regression model. The modulatory potential of psychological distress on compliance will be assessed using an interaction term between patient anxiety/depression and HRQoL in a logistic regression model.

An analysis of missing HRQoL data profiles will also be performed. If a missing not at random (MNAR) profile is demonstrated/suspected, multiple imputation of missing data can be performed in sensitivity analysis, taking into account the variables associated with the occurrence of missing data.

The significance level for the statistical analyses is fixed at a *P* value of less than 0.05, and all tests will be bilateral. The data analysis will be performed by using Statistical Analysis System (SAS) version 9.4 (SAS Institute Inc., Cary, NC, USA).

#### 2.6.3. Sensitivity analysis

Contamination-based intent-to-treat analysis will be conducted to account for potential cross-contamination between the arms.

### 2.7. Ethical considerations

As a specific research intervention is carried out, the protocol falls within the scope of interventional biomedical research and was authorized by the French Health Agency (ANSM- Agence Nationale de la Sécurité du Médicaments et des produits de santé”, IDRCB number: 2019-A01323–54) in June 2019 and by the French Ethical Research Committee (CPP- “Comité de Protection des Personnes”) in October 2019. The clinical trial has been registered at ClinicalTrials.gov with the identifier NCT04176809.

This study will be carried out in accordance with the ethical principles of the Helsinki Declaration and the Good Clinical Practice of the International Harmonization Conference. Participants must provide informed consent. Subjects will be informed of the objectives of the project and the risks and benefits of the explorations to be carried out. Confidentiality of participant data will be guaranteed at all times in agreement with the CNIL MR01 reference methodology registered for CGFL (1878714v0, 30/07/2015).

## 3. Discussion

This study will evaluate the usefulness of systematic assessment of HRQoL coupled with therapeutic information to enhance compliance with ET in patients with non-metastatic BC. To take into account that compliance decreases over time, a reminder to encourage patients to take ET was included in the intervention and will be sent every month. A steering committee has also been set and will be responsible for all decisions concerning the study.

In the future, if results are positive, this intervention will be implemented in clinical practice; therefore, we have chosen to be as close as possible to routine clinical practice. Routinely, women with HR^+^ BC treated with ET will have a consultation two or three times a year. HRQoL will be assessed before each consultation and will be monitored three times during study duration.

This routine HRQoL assessment would enable clinicians to identify and better understand the areas in which patients who receive ET most need support, therefore guiding patient individual management. Systematic assessment of HRQoL could provide an additional endpoint for measuring patient status and treatment-related symptoms, including ET.

If study results are positive, electronic assessment of HRQoL coupled with therapeutic information could be offered in daily clinical practice as an integral part of the care process for patients who receive ET. Moreover, this intervention could be generalized to other centers in France given that every cancer center has a service dedicated to therapeutic information.

## 4. Trial status

Protocol version 1.1. Date: Aug. 20, 2019. Recruitment began in January 2020 and will be completed by around December 2022.

## Data Availability

The data that support the findings of this study are available from the corresponding author upon reasonable request.

## Abbreviations

AI: Aromatase inhibitor
BC: Breast cancer
CHES: Computer-based Health Evaluation System
EORTC: European Organization for Research and Treatment of Cancer
ET: Endocrine therapy
FACT-G: Functional Assessment of Cancer Therapy - General
HADS: Hospital Anxiety and Depression Scale
HR^+^: Hormone receptor–positive
HRQoL: Health-related quality of life
MGL: Morisky Green Levine
SSQ6: Sarason’s Social Support Questionnaire
